# Supplementary material for: HLA-A01 and HLA-B27 Supertypes, but Not HLA Homozygocity, Correlate with Clinical Outcome among Patients with Non-Small Cell Lung Cancer Treated with Pembrolizumab in Combination with Chemotherapy
Source: Cancers (Basel). 2024 Sep 7;16(17):3102. doi: 10.3390/cancers16173102 (PMC11394546; doi:10.3390/cancers16173102)
Supplement: Supplementary file 1 [file cancers-16-03102-s001.zip › Supplementary Table S1.pdf]

Supplementary Table S1: Frequency of HLA-I supertypes among patients treated with immunotherapy alone versus chemoimmunotherapy.

**HLA-I supertype**	**NSCLC patients treated with**		**P value**
	**IO alone (n=161)***	**IO/Chemo (n=53)**	
**HLA-A24**			
+ve	38 (23.6)	12 (22.6)	0.524
-ve	123 (76.4)	41 (77.4)	
**HLA-A01**			
+ve	71 (44.1)	20 (37.7)	0.258
-ve	90 (55.9)	33 (62.3)	
**HLA-A02**			
+ve	80 (49.7)	26 (49.1)	0.531
-ve	81 (50.3)	27 (50.9)	
**HLA-A03**			
+ve	84 (52.2)	21 (39.6)	0.077
-ve	77 (47.8)	32 (60.4)	
**HLA-B58**			
+ve	13 (8.1)	0	0.022#
-ve	148 (91.9)	53 (100)	
**HLA-B62**			
+ve	30 (18.6)	12 (22.6)	0.325
-ve	131 (81.4)	41 (77.4)	
**HLA-B27**			
+ve	32 (19.9)	15 (28.3)	0.138
-ve	129 (80.1)	38 (71.7)	
**HLA-B44**			
+ve	90 (55.9)	28 (52.8)	0.408
-ve	71 (44.1)	25 (47.2)	
**HLA-B07**			
+ve	75 (46.6)	23 (43.4)	0.404
-ve	86 (53.4)	30 (56.6)	
**HLA-B08**			
+ve	37 (23)	12 (22.6)	0.561
-ve	124 (77)	41 (77.4)	

HLA: Human Leukocyte Antigen, *cohort from Abed et al 2020 [10], # the frequency of 0 make the P value of less significance.
