# Supplementary figures and images for: HLA-A01 and HLA-B27 Supertypes, but Not HLA Homozygocity, Correlate with Clinical Outcome among Patients with Non-Small Cell Lung Cancer Treated with Pembrolizumab in Combination with Chemotherapy
Source: Cancers (Basel). 2024 Sep 7;16(17):3102. doi: 10.3390/cancers16173102 (PMC11394546; doi:10.3390/cancers16173102)

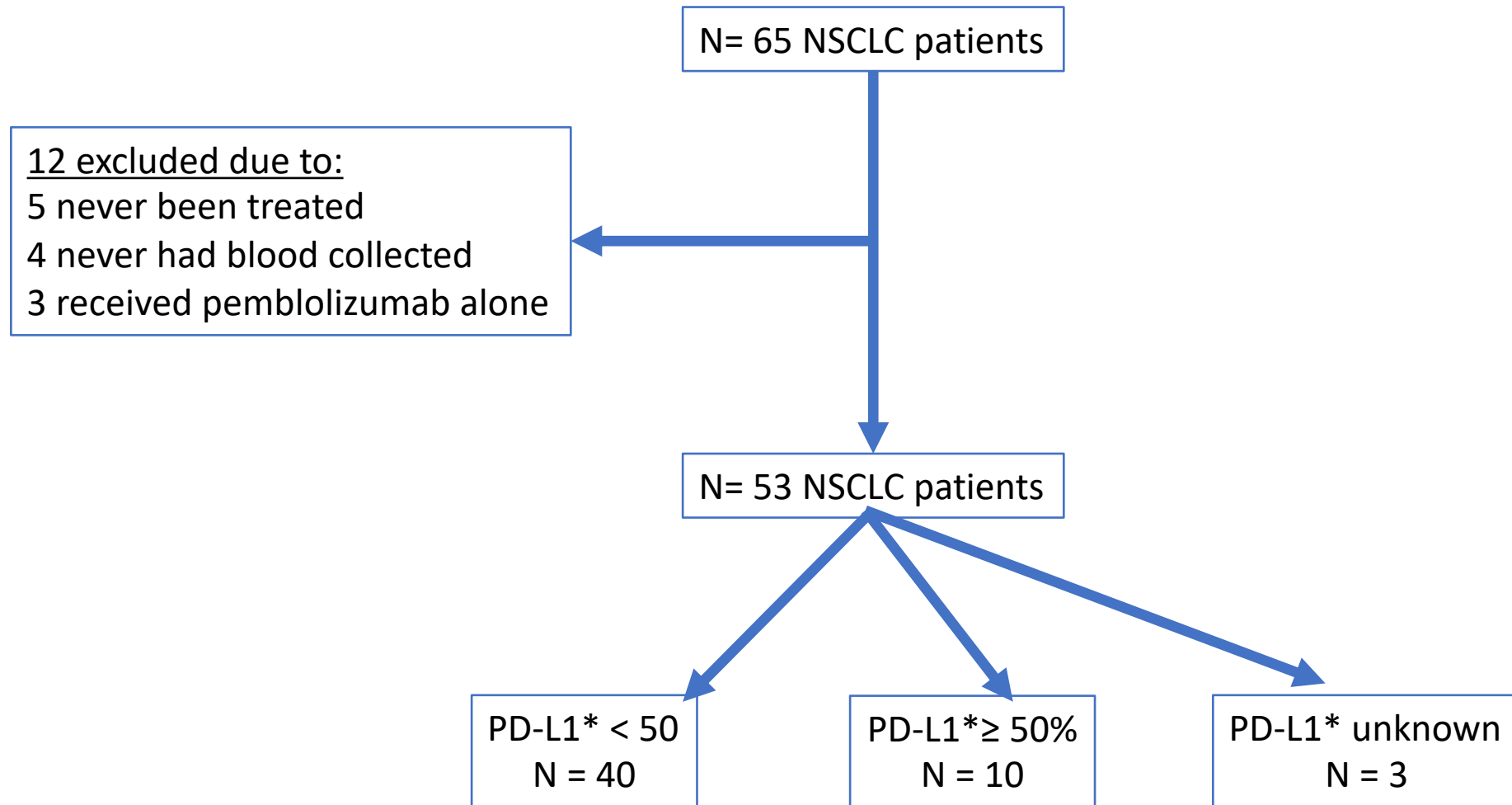

\*DAKO 22C3 CLONE

Supplement: Supplementary file 1 [file cancers-16-03102-s001.zip › Supplementary Figure S1.pdf]
